# Prevalence of Ethical Issues in Patients with Advanced Cancer: Secondary Analysis of Pooled Data from the Development and Validation Cohorts of the PALCOM Scale for the Complexity of Palliative Care Needs

**DOI:** 10.3390/cancers17081345

**Published:** 2025-04-16

**Authors:** Albert Tuca, Margarita Viladot, Carmen Barrera, Manoli Chicote, Teresa Gabarró, Gemma Carrera, Anais Pascual, Elena Font, Elena Angulo, Ester Hernández-Godoy, Javier Marco-Hernández, Lucia Llavata, Joan Padrosa, Carles Zamora-Martínez, Nuria Codorniu

**Affiliations:** 1 Unit of Supportive Care in Cancer, Medical Oncology Department, Hospital Clinic and Translational Genomics and Targeted Therapies in Solid Tumours, August Pi i Sunyer Biomedical Research Institute, IDIBAPS, University of Barcelona, 08036 Barcelona, Spain; viladot@clinic.cat (M.V.); mbarrera@clinic.cat (C.B.); mchicote@clinic.cat (M.C.); gabarro@clinic.cat (T.G.); gecarrera@clinic.cat (G.C.); alpascual@clinic.cat (A.P.); efont@clinic.cat (E.F.); jmarco@clinic.cat (J.M.-H.); llavata@clinic.cat (L.L.); padrosa@clinic.cat (J.P.); czamora@clinic.cat (C.Z.-M.); 2 Psychosocial Support Team, “La Caixa” Foundation (EAPS), Clinic Hospital of Barcelona, 08036 Barcelona, Spain; anguloa@clinic.cat (E.A.); eshernandez@clinic.cat (E.H.-G.); 3 University Chair in Palliative Care, University of Barcelona, 08036 Barcelona, Spain; 4 Foundation for Dependency Care, Sant Joan de Deu, 08014 Barcelona, Spain; nuria.codorniu@sjd.es

**Keywords:** advanced cancer, ethic issues, complexity of palliative care needs, early palliative care

## Abstract

Introduction: The life experience of patients with advanced cancer and limited life expectancy is unique and profoundly complex, often leading to moral discrepancies among the various individuals involved in decision making. There are no data in the literature on the prevalence of ethical issues in the end-of-life care of patients with advanced cancer. Objectives: The primary objective of this study is to identify the overall and specific prevalence of ethical issues in the end-of-life care process for patients with advanced cancer. Method: We performed a secondary analysis using pooled data from the prospective development and validation cohorts of the PALCOM scale, focusing on the complexity of palliative care needs. This was performed to determine the overall and specific prevalence of ethical issues, describe their evolution over a 6-month follow-up period, and analyze their association with the level of palliative care complexity. Results: A total of 607 patients with advanced cancer and a life expectancy of 6 months or less were included. The consistency in clinical data and the frequencies of the PALCOM scale domains between the development and validation cohorts, conducted in various settings and times, confirmed the reliability of the pooled data sample. Systematic application of the PALCOM scale identified 126 patients (20.7%) who experienced at least one ethical issue. A total of 204 ethical issues (1.6 per patient) were recorded, related to the following: the proportionality of healthcare intervention (15.6%); information (13.0%); research (2.9%); the desire to hasten death (1.8%); and palliative sedation (0.15%). The monthly probability of presenting an ethical issue was significantly higher at the baseline visit (24.0%) compared to the rest of the 6-month follow-up period (14–17%) (*p* < 0.001). The prevalence of ethical issues was notably higher in patients with greater palliative care complexity as measured by the PALCOM scale: 4.5% in low complexity, 19.5% in medium complexity, and 30.8% in high complexity (*p* < 0.001). Conclusions: The prevalence of ethical issues in patients with advanced cancer is high. Most of these issues are directly or indirectly related to the preservation of patient autonomy in the decision-making process. The presence of ethical issues is significantly associated with the greater complexity of palliative care needs. In this context, it is crucial for healthcare professionals to strengthen both communication skills and basic competencies to effectively identify, assess, and manage these ethical issues.

## Simple Summary

The experience of individuals with incurable cancer and a limited life expectancy is profoundly complex. Ethical issues arise when the commonly accepted guiding principles of healthcare conflict or are interpreted differently by the various individuals involved in the decision-making process in a specific situation. Our study, based on a pooled analysis of two prospective cohorts of advanced cancer patients, confirmed a high prevalence of patients facing ethical issues. Most of these issues were primarily related to the proportionality of healthcare interventions, patient information, or the desire to hasten death. It is important to highlight that ethical issues were associated with a greater complexity of palliative care needs, and their prevalence decreased after the baseline visit, suggesting that early identification and intervention can improve outcomes. Ethical issues can cause significant distress for patients, their families, and the care team. All the issues observed in this study were directly or indirectly related to decision making and the recognition of patient autonomy. Based on these findings, it is crucial to strengthen basic competencies in clinical ethics and communication skills among healthcare professionals. This approach would enable the early identification of ethical issues, facilitate measured interventions, and improve supportive care outcomes.

## 1. Introduction

Bioethics is defined as the systematic study of the moral dimensions of life sciences and healthcare. This definition acknowledges the inherent moral nature of actions, behaviors, and health policies [1].

The paradox of cancer treatment in the 21st century is that, despite significant advances in prevention, diagnosis, and high-precision treatments, providing comprehensive care for patients with incurable cancer remains a challenge. Conversations between professionals and patients with advanced cancer about the value and meaning of the natural process of death, when it is unavoidable, can be particularly difficult [2]. The life experience of patients with advanced cancer and limited survival expectancy is unique and profoundly complex. Symptomatic burden, functional decline, emotional impact, family support, social environment, spirituality, moral values, life history, cultural context, and the conditions of the patient’s care setting significantly influence the coping strategy for the end-of-life process [2,3,4,5]. It is a complex process because it depends on the continuous and non-linear interaction of multiple multidimensional variables, maintaining a frequently unstable balance with not always predictable outcomes [5,6,7,8,9,10,11,12,13].

In the complex end-of-life scenario, the patient, family, and healthcare professionals actively participate in the decision-making process. Healthcare professionals provide objective information about the disease and its treatment, based on scientific evidence. However, each patient assigns a unique and subjective value and meaning to their illness. This dynamic and inter-subjective process often leads to moral discrepancies among the various individuals involved in decision making.

Ethical reflection emerges when the commonly accepted codes, rules, or habits guiding the decision-making process become uncertain. The widely accepted ethical principles of beneficence, autonomy, justice, and non-maleficence govern the actions of healthcare professionals [14]. Ethical issues arise when, in a specific situation, one of these principles is violated, conflicts with another, or is interpreted differently by the various individuals involved.

Published studies focus on the narrative description of ethical issues in the end-of-life process from a theoretical perspective or detail, through surveys, the most common ethical concerns among professionals [15,16,17,18,19,20,21,22,23]. However, the literature does not provide data on the prevalence of ethical issues in the end-of-life process in real clinical practice. In 2021, our group published the only prospective study on the prevalence of ethical dilemmas in the end-of-life process of patients with advanced cancer. The study, which systematically evaluated all patients in the prospective cohort for developing the PALCOM scale for palliative care complexity, found that 27.8% of patients experienced ethical issues [13].

The PALCOM scale is a multidimensional assessment tool designed to measure the complexity of palliative needs in patients with advanced cancer. This scale comprises five assessment domains, including the identification of ethical or existential issues. In 2018, our group published a prospective cohort study that led to the creation and development of the PALCOM scale (development cohort) [9]. In 2021, we published the aforementioned secondary analysis on the prevalence of ethical issues, based exclusively on the development cohort [13]. In 2023 and 2024, we published two validation studies that used the same inclusion criteria and assessment system (validation cohort) [10,11]. These studies demonstrated the PALCOM scale’s strong predictive accuracy in assessing the complexity of palliative care needs, with an F1 score of 0.81 and an area under the ROC curve of 0.907. We believe that now is an opportune moment to conduct a pooled analysis of data from the development and validation cohorts of the PALCOM scale. This analysis will help reinforce the consistency of prevalence data on ethical issues.

The objective of this study is to determine the overall and specific prevalence of different ethical issues observed in the end-of-life process in patients with advanced cancer. Our hypothesis is that the prevalence of ethical issues is high, that there are no significant differences between the values observed by different teams and in different study periods (development and validation cohorts), and that the overall prevalence decreases during follow-up, likely because early detection allows for specific intervention.

## 2. Materials and Methods

We performed a secondary analysis of pooled data from the development and validation cohorts of the PALCOM scale, which evaluates the complexity of palliative care needs in patients with advanced cancer. Both cohorts were prospective and multicenter, with a follow-up period of 6 months. They applied the same inclusion criteria and used the same methodology for both the assessment and the recording of variables.

### 2.1. Setting and Study Periods

The development cohort, aimed at constructing a model for the complexity of palliative care needs (PALCOM scale), included patients from November 2012 to January 2013. The validation cohort, aimed at confirming the consistency and predictive value of the PALCOM scale, included patients from December 2020 to April 2021. Both studies involved multiple public healthcare centers across all levels of care in the Autonomous Community of Catalonia (Spain): primary care, hospitals, hospital and home palliative care (PC), and medium–long-stay units. The development cohort included 24 healthcare centers (16 primary care, 3 hospitals, 3 home palliative care teams, and 2 medium–long-stay centers). The validation cohort included 7 healthcare centers (1 primary care, 2 hospitals, 3 home PC teams, and 1 medium–long-stay center).

**Figure 1:**
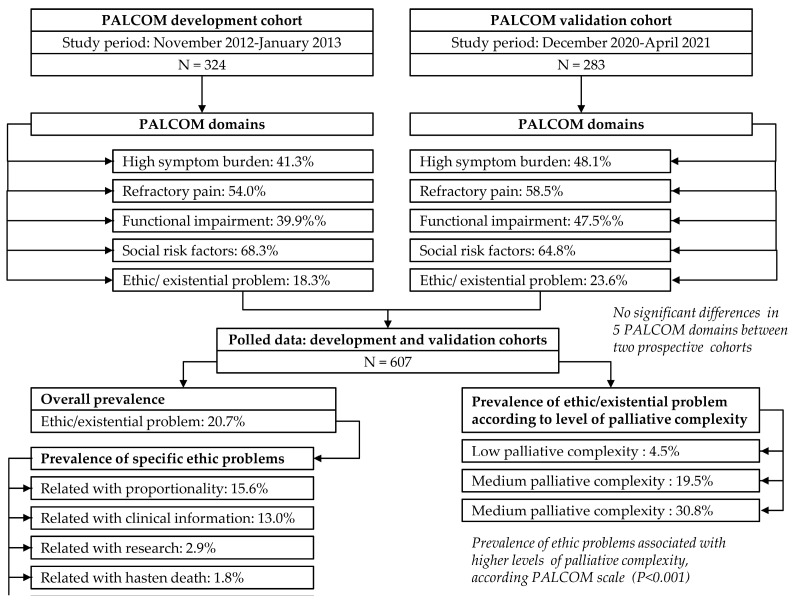
Flow diagram of pooled data from PALCOM cohorts: prevalence of ethical issues in advanced cancer patients.

The results from the development and validation cohorts, along with a secondary analysis of the prevalence of ethical issues based solely on the development cohort, were published in 2018, 2023, and 2021, respectively [9,10,11,13]. The current study presents a secondary analysis of the prevalence of ethical issues using pooled data from both cohorts. The objectives are to increase sample size and consistency, compare results between the two cohorts, and analyze their evolution during follow-up (Figure 1).

### 2.2. Sample

In this study, all patients visited by the treating team who met the following inclusion criteria were consecutively recruited: age ≥ 18 years, diagnosis of advanced cancer, estimated life expectancy ≤6 months, and signed informed consent.

### 2.3. Design

This analysis is based on prospective multicenter observational cohort studies involving patients with advanced cancer (development and validation cohorts of the PALCOM scale), with a maximum follow-up period of 6 months.

### 2.4. Objectives

The main objective of this study was to determine the overall prevalence of ethical issues in patients with advanced cancer. The secondary objectives were (a) to identify the prevalence of different sub-categories of ethical issues; (b) to compare the prevalence between the two cohorts and assess their consistency; and (c) to describe the evolution of these issues over the 6-month follow-up period of the study.

### 2.5. Instruments

The analysis was based on two specific instruments: the PALCOM scale and the operational definition of ethical problems.

#### 2.5.1. PALCOM Scale of Palliative Complexity

The PALCOM scale is a predictive tool designed to assess the complexity of palliative care needs. It comprises five multidimensional domains: (a) symptomatic burden, (b) refractory pain, (c) functional decline, (d) socio-family risk factors, and (e) ethical issues (Table 1). Each domain is scored dichotomously, with 0 indicating the absence and 1 indicating the presence of the corresponding variables [9,10,11]. The final score is the sum of the values across all domains. Patients scoring 4–5 are classified as high complexity and require systematic, intensive intervention by multidisciplinary palliative care (PC) teams, whether shared with referring teams or not. Those scoring 2–3 are classified as medium complexity and need systematic intervention by multidisciplinary PC teams, at an intensity adjusted to their needs, whether shared with referring teams or not. Patients scoring 0–1 are classified as low complexity and generally do not require systematic intervention by multidisciplinary PC teams, except for occasional consultations.

#### 2.5.2. Definition of Ethical Issues

The identification of ethical issues can depend on the individual interpretation of observers. Therefore, the research team agreed on the following operational definition for both cohorts: a discrepancy in shared decision making in a specific situation where it is necessary to choose between opposing options, morally or existentially acceptable from the individual perspective of the different participants in the deliberation. Ethical issues observed were categorized based on the sub-categories identified in the analysis of the fifth domain of the PALCOM scale within the development cohort [9]: (a) related to information; (b) related to the proportionality of therapeutic and supportive measures (discrepancies in proportionality among family members, between the patient/family and the care team, and among care team members); (c) related to research and clinical trials; (d) related to palliative sedation; (e) related to the desire to hasten death; and (f) other unspecified issues.

### 2.6. Data Collection

All descriptive variables of the study, PALCOM domains, and ethical issues were identified and recorded by the patient’s treating team within the context of their daily clinical practice. Despite the advanced competencies in palliative care possessed by most professionals participating in the study, all received specific training on the PALCOM scale and the consensus definition of ethical issues to optimize the accuracy of study records.

#### 2.6.1. Main Outcome Variable

The main variable in this study was the prevalence of ethical issues in patients with end-of-life cancer at the baseline visit. The prevalence of ethical issues was evaluated both globally and broken down into the aforementioned sub-categories (information, proportionality, research, palliative sedation, and miscellaneous) at the baseline visit and monthly during follow-up.

#### 2.6.2. Descriptive Variables

The following descriptive and clinical variables were recorded and analyzed: age, gender, primary cancer origin, survival, place of death, level of palliative care complexity, and PALCOM scale domains, which were (a) high symptom burden (≥5 symptoms of at least moderate intensity in a systematic 10-symptom record based on the Edmonton Symptom Assessment Scale model [24]); (b) risk criteria for refractory pain according to the Edmonton Classification System for Cancer Pain [25,26,27]; (c) Karnofsky Performance Status; and (d) socio-familial risk factors.

#### 2.6.3. Method for Collecting Data

All study variables were recorded directly by the treating professional in an electronic data collection notebook, coded to ensure the confidentiality of participants’ personal data. No individuals outside the research project had access to the coded database.

### 2.7. Sample Size and Statistical Analysis

The sample size estimation was based on the maximum recruitment capacity of the participating centers, ensuring the integration of the registry of variables into their daily practice. Consequently, it was estimated that each of the two cohorts should include at least 250 patients, with 40% classified as medium–high complexity. Following the rule of at least 10 events per independent factor, a multivariate logistic regression analysis could be constructed with more than 10 factors, identifying odds ratios with effects ≥ 1.10 [9,11].

The prevalence of patients with ethical issues and the frequencies of sub-categories were assessed both overall and stratified by the level of complexity on the PALCOM scale and by each month of follow-up. The prevalence observed in the development and validation cohorts were compared, considering that they were conducted at different times and by different care teams. Categorical and dichotomous variables were analyzed using absolute and relative frequencies, while continuous variables were described by calculating the mean and standard deviation (SD) with a 95% confidence interval. Fisher’s exact test and the non-parametric Mann–Whitney–Wilcoxon test were employed to compare variables based on complexity levels.

### 2.8. Ethical Considerations

This study was conducted in accordance with the Declaration of Helsinki and was approved by the Clinical Research Ethics Committee of the Hospital Clínic de Barcelona, University of Barcelona (code: HCB/2016/0611, 19 September 2016). The management of the obtained information was carried out in accordance with Spanish data protection laws.

## 3. Results

The pooled analysis included a total of 607 patients, with 324 (53.4%) from the development cohort and 283 (46.6%) from the validation cohort. Monthly follow-up data were available only for the validation cohort, as this variable was not recorded in the development cohort.

In the pooled data, the average age of the patients was 70 years (SD ± 11), with 350 men (57.7%). A total of 355 patients (58.5%) were included from hospitals, while 255 (41.5%) were from community health centers. The most common primary cancer origins were lung (23.1%), colon (15.5%), prostate (7.6%), and breast (6.8%). A total of 500 patients had metastatic disease (82.4%), and 107 had advanced loco-regional disease (17.6%). At the time of inclusion, 462 (76.1%) patients were undergoing cancer treatment. The most common symptoms recorded in this study were asthenia (93.6%), pain (80.7%), anorexia (78.9%), anxiety (69.5%), sadness (69.4%), and insomnia (60.6%). The frequencies of the five PALCOM scale domains were (a) high symptom burden 44.5%, (b) risk criteria for refractory pain 56.2%, (c) Karnofsky Performance Status ≤60% 43.5%, (d) socio-familial risk factors 66.7%, and (e) ethical issues 20.7%. Three hundred seventy-nine patients (62.4%) died before the end of the maximum follow-up period of 6 months. It is noteworthy that no significant differences were observed between the two cohorts regarding sociodemographic outcomes, primary cancer origin, functional status, symptom prevalence, and the five domains of the PALCOM scale (Table 2 and Table 3). This finding underscores the consistency and reliability of the evaluation method, given that the two studies were conducted at different times by distinct healthcare teams.

### 3.1. Prevalence of Ethical Issues

In the pooled data, 126 patients (20.7%) presented at least one ethical issue (59 from the development cohort and 67 from the validation cohort) (Table 4). A total of 204 ethical issues were recorded (117 in the development cohort and 87 in the validation cohort). Therefore, the average number of ethical issues per patient was 1.6 (1.9 in the development cohort and 1.3 in the validation cohort). No significant differences were observed between the two cohorts.

In the pooled data, 79 patients (13.0%) presented issues related to information. This was slightly more frequent in the development cohort (15.7%) than in the validation cohort (10.0%), but the difference was not statistically significant. In the pooled data, 95 patients (15.6%) presented issues related to the proportionality of therapeutic or supportive measures, with no significant differences between the development cohort (16.7%) and the validation cohort (14.5%). The discrepancies in proportionality were (a) among different family members in 47 patients (7.7%); (b) between the patient or their family and the healthcare team in 27 patients (4.4%); and (c) among different members of the healthcare team in 25 patients (4.1%). No significant differences were observed between the frequencies in the development and validation cohorts. In the pooled data, 18 patients (2.9%) presented issues related to research, with no significant differences between the development cohort (2.5%) and the validation cohort (3.5%). In the pooled data, 11 patients (1.8%) explicitly, consistently, and repeatedly expressed the desire to hasten death, with no significant differences between the development cohort (1.2%) and the validation cohort (2.4%).

The high prevalence of ethical problems observed highlights the importance of their systematic assessment. The absence of differences in the overall prevalence and sub-categories of ethical issues between the two cohorts reinforces the consistency of the agreed-upon definition of ethical issues.

### 3.2. Evolution During Follow-Up

**Figure 2:**
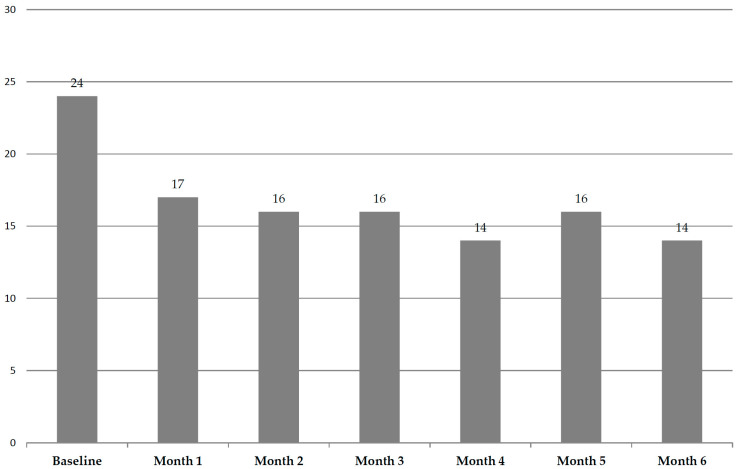
Overall frequencies of ethical issues during follow-up in validation cohort (%). The prevalence of ethical issues at baseline was significantly higher than that observed in the subsequent follow-up months (*p* < 0.001).

The monthly probability of presenting ethical issues during the 6-month follow-up was evaluable only in the validation cohort. A total of 67 patients (23.7%) presented ethical issues at the baseline visit. Among the patients who were alive in the first month of follow-up, 44 (17.1%) presented ethical issues, and 32 (15.9%) presented them in the second month, 25 (15.9%) in the third month, 18 (14.1%) in the fourth month, 18 (15.8%) in the fifth month, and 14 (14.4%) in the sixth month (Figure 2). The probability of presenting ethical issues was significantly higher at the baseline visit (*p* < 0.001), with no significant differences in this monthly probability from the first month of follow-up onwards. This interesting finding suggests that the early detection of ethical issues within the context of a systematic evaluation can facilitate their management, reduce their frequency, and thus improve the outcome of care.

### 3.3. Prevalence of Ethical Issues According to Palliative Complexity Level on the PALCOM Scale

The PALCOM scale determines the level of palliative complexity based on the interaction of five assessment domains: symptom burden, potentially refractory pain, functional impairment, socio-familial risk, and ethical issues. Cases with 4–5 affected domains are considered highly complex, 2–3 domains indicate medium complexity, and 0–1 domain corresponds to low complexity.

In the pooled data, 118 patients (19.5%) were classified as having low palliative complexity, 306 (50.5%) as medium, and 182 (30.0%) as high. Higher complexity levels are significantly associated with increased six-month mortality rates (low 43.2%, medium 62.7%, and high 75.8%) (*p* < 0.001) and a higher probability of in-hospital death (low 7.6%, medium 16.0%, and high 28.6%) (*p* < 0.001). The likelihood of encountering ethical issues is significantly higher at greater levels of palliative complexity according to the PALCOM scale (*p* < 0.001): low 4.2%, medium 19.5%, and high 30.8% (Table 3).

## 4. Discussion

We consider that combining data from both the development and validation cohorts of the PALCOM scale is beneficial for expanding the study sample and enhancing the reliability of the preliminary findings on the prevalence of ethical issues, which were originally derived only from the development cohort.

The data provided correspond to two prospective cohorts of patients with advanced cancer, conducted with the same inclusion and evaluation system by different teams at different times. We believe that the absence of statistically significant differences between socio-demographic and outcome variables in the comparison of both cohorts confirm the consistency of the aggregated analysis and the absence of inclusion or evaluation biases.

### 4.1. Overall Prevalence of Ethical Issues

In the pooled data, one-fifth of patients presented at least one ethical issue, according to the treating professionals. It is noteworthy that many of these patients simultaneously presented multiple ethical issues (an average of 1.6 ethical issues per patient). It is clear that ethical issues can cause significant discomfort for all parties involved in their discussion. The first step in addressing such a problem is its identification, and the second is to manage the conflict explicitly and respectfully. Conflict management skills can be essential for health professionals in resolving these issues. In fact, it is noteworthy that the prevalence of ethical issues in this study was significantly higher at the baseline visit compared to the prevalence observed during the subsequent six months of follow-up. These data suggest that early identification and intervention can reduce the frequency of ethical issues and, therefore, improve the outcomes of patient support measures. Overall, we consider these findings to be novel and relevant due to the high observed prevalence and the lack of previously published data in this area.

### 4.2. Prevalence of Different Categories of Ethical Issues

In this study, ethical issues were classified into five different categories: information, proportionality, research, palliative sedation, and the desire to hasten death. This classification was agreed upon by the research team of the development and validation cohorts of the PALCOM scale. It was based on multiple expert opinion publications that described the theoretical aspects of the issues or surveys that identified the main ethical concerns of healthcare professionals in the end-of-life process [15,16,17,18,19,20,21,22,23]. Interestingly, all 214 ethical issues detected in the aggregated data of this study could be included in one of these predetermined categories.

#### 4.2.1. Ethical Issues Related to Information

Communication between a doctor and a patient is a process of information exchange aimed at providing the patient with the necessary knowledge to make reflective and autonomous decisions in a specific clinical context. It is an asymmetric and bidirectional process. The healthcare professional, who has knowledge of the clinical data, must explain and advise the patient clearly, truthfully, comprehensibly, and personally about everything concerning their health and must recognize the value and meaning the patient attributes to the received information [28,29,30]. The available literature confirms that adequate communication can improve adherence to proposed treatments, quality of life, and patient satisfaction, as well as reduce uncertainty and emotional impact [31,32,33,34,35,36,37,38,39,40,41,42,43,44,45].

In the complex process of informing about the diagnosis, extent, treatment options, and prognosis of cancer, ethical issues may arise. These issues are related to both the quality and quantity of the information provided, which is often modulated according to the professional’s criteria in response to the patient’s or family’s reaction, as well as the timing and context in which the information is offered. The healthcare professional must manage multiple and unpredictable coping styles to the life-threatening nature of the disease, where the patient’s expectations and hopes are not always realistic and where there may be tendencies towards the compassionate limitation of information by the family.

In this study, the ethical issues related to information that complicated the decision-making process were as follows: (a) information exceeding the patient’s desired limits; (b) adaptive denial; and (c) conspiracy of silence. Information exceeding the patient’s desired limits was described as that which caused a high emotional impact and hindered their participation in decision making because it was provided rapidly in all its aspects without respecting the patient’s opinion on the amount, timing, or context in which it was given. Denial was described as an adaptive process in which the patient, implicitly or explicitly, preferred not to know all aspects of their disease, especially the prognosis, or adopted alternative arguments to avoid recognizing a reality they felt was unmanageable. The conspiracy of silence was defined as the explicit agreement of the family to avoid providing information, especially the prognosis, with a compassionate intention based on the belief that the patient does not have sufficient emotional resources to face the life-threatening situation.

Various studies indicate that between 20% and 69% of patients with advanced and incurable cancer are unaware of both the prognosis and the palliative intent of chemotherapy or radiotherapy treatment [40,46,47,48,49,50]. Communication with the patient is an ethical and clinical imperative for all healthcare professionals. If a patient has insufficient information, it is the healthcare professional’s duty to expand it truthfully, comprehensibly, and personally to ensure autonomy in decision making. In this study, the prevalence of ethical issues related to information was limited to situations where the previously provided information or the adaptive reaction to it generated an ethical issue that hindered shared decision-making with the patient. This is likely the reason for the observed differences between the general record on the lack of knowledge about the disease prognosis mentioned earlier (20–69%) and the data provided by this study.

In 2018, the healthcare ethics consultation service at Memorial Sloan Kettering Cancer Center in New York published a retrospective study on the prevalence of ethical issues consulted. According to this study, 8% of these consultations were related to communication problems and 13% to cases of denial by the patient or their family [51].

#### 4.2.2. Ethical Issues Related to Proportionality

Deliberation on the proportionality of healthcare interventions caused an ethical issue in 16% of the cases included in this study. The observed issues focused on two distinct situations: futility and treatment refusal. Futility is defined as a medical act considered useless or disproportionate due to its unlikely efficacy and the lack of reasonable benefit in an individual clinical condition, according to available scientific evidence [52,53]. Refusal of treatment that healthcare professionals consider appropriate requires careful deliberation but is not ethically disputable if it is based on the patient’s reflective reasoning and the conditions of their autonomy of will are met (cognitive competence, complete information, and absence of external interferences) [54,55,56].

In this study, most discrepancies regarding proportionality were observed among the patient’s family circle. Less frequently, proportionality conflicts were recorded between the patient/family and the healthcare team or among the different healthcare professionals involved in the patient’s care. Discrepancies regarding the withdrawal of life support were observed in very few cases.

In the previously mentioned study by the Memorial Sloan Kettering Cancer Center in New York, 22% of the consultations to the healthcare ethics service were related to the futility of healthcare interventions, 5% to treatment refusal, and 2% to the withdrawal of life support. Additionally, they observed that in 11% of these consultations, there were discrepancies among the patient’s family members: in 3% between the patient/family and the healthcare team and in 5% among the members of the treating team [51].

#### 4.2.3. Ethical Issues Related to Research

Research in patients with advanced cancer who have not responded to conventional treatments undeniably represents an ethical challenge. The vulnerability of these patients and the desperate need to find therapies that delay the progression of their disease can lead to the misinterpretation of information, affect the informed consent process, and encourage the acceptance of inclusion in an early-phase clinical trial [57,58,59,60,61].

In this study, ethical issues related to research were detected at a low but significant frequency. As expected, all patients participating in clinical trials received verbal and written information and signed an informed consent form. However, researchers identified that some of these patients’ expectations were not fully aligned with the actual expected outcomes, or they accepted inclusion in the trial out of fear of being abandoned by the healthcare system. It is noteworthy that conflicts related to research represented 2% of the consultations to the healthcare ethics service at Memorial Sloan Kettering Cancer Center in New York [51].

#### 4.2.4. Ethical Issues Related to Palliative Sedation

Palliative sedation is defined as the deliberate administration of sedatives in the necessary doses and combinations to reduce the level of consciousness of a terminal patient for the time required to adequately relieve one or more refractory symptoms [62,63,64]. Approximately 25% (10–50%) of patients with advanced cancer require palliative sedation in the final days of life [64].

Both the ethical aspects and procedures for the application of terminal sedation, as well as the differential criteria with euthanasia, are widely accepted. However, in daily clinical practice, dilemmas can occasionally arise related to the interpretation of the ethical requirements of palliative sedation: (a) the definition of refractory symptoms; (b) the adequacy of sedative doses proportional to the goal of reducing the level of consciousness, not shortening life span; and (c) the involvement of the patient or their family in the sedation decision (explicit, implicit, or delegated consent).

Currently, ethical issues related to palliative sedation are infrequent. In fact, in this study, only one case was detected where the decision for palliative sedation was ethically complex. In 2016, a study based on a survey of professionals was published to determine the frequency and difficulty of resolving 20 previously agreed-upon ethical dilemmas in the end-of-life process. The analysis of the responses concluded that the frequency and difficulty of ethical issues related to palliative sedation were low, ranking 18th among the 20 ethical dilemmas examined [16]. In the study by the healthcare ethics service at Memorial Sloan Kettering Cancer Center in New York, no consultations related to palliative sedation were recorded [51].

#### 4.2.5. Ethical Issues Related to the Desire to Hasten Death

In the context of incurable cancer, some patients may wish to end their lives. The desire to hasten death is a complex phenomenon influenced by various dimensions of human suffering (physical, emotional, social, and spiritual) [65,66,67,68,69]. In some cases, intolerable and refractory physical or psychological suffering may be the determining factor. In others, it may be physical dependency, unwanted loneliness, a lack of social support, or the feeling of being a burden to others. It can also appear in patients who have serenely accepted death and consider the remaining time to live as useless and painful.

In this study, the desire to hasten death was recorded as the idea of not wanting to continue living in conditions that the patient considered intolerable and that violated their concept of personal dignity, argued reflectively and repeatedly. Therefore, patients who occasionally expressed the desire to die in the context of a specific crisis of physical or psychological suffering, or those who expressed it as a symptom of major depression, were not included. Identification was carried out in the context of the study’s systematic multidimensional evaluation according to the clinical judgment of the treating professional, without using any instrument specifically designed to detect the desire to hasten death.

The prevalence of the desire to hasten death in this study was low. The available literature on the prevalence of the desire to hasten death is very heterogeneous, ranging from 1.5% to 35%, depending on the detection instrument used, the agreed cutoff point, and the care setting [67,68,69].

In recent years, euthanasia has been a topic of social debate in many developed countries. The core of the discussion is whether a person suffering from an incurable and progressive disease that causes intolerable and refractory physical or psychological suffering can decide when, how, and where to die. It is a discussion that seeks to harmonize the right to life and the right to freedom and dignity from a pluralistic and possibilistic perspective. Seven countries worldwide have approved laws regulating euthanasia [70]. In Spain, where this study was conducted, euthanasia was legalized in March 2021 [71]. It is noteworthy that the study presented was carried out before this law came into effect (June 2021). We do not know if the implementation of this law will influence the free and spontaneous expression of the desire to hasten death.

### 4.3. Ethical Aspects and Palliative Complexity

Complex systems are characterized by the interaction of numerous variables in an unstable equilibrium, making them highly sensitive to initial conditions and often resulting in unpredictable outcomes [3,4,5,6,7,8].

The PALCOM scale identified five domains (symptom burden, refractory pain, functional deterioration, social risk, and ethical issues) whose continuous interaction determined the level of complexity of palliative needs in patients with advanced cancer in real clinical practice. Higher complexity levels were associated with high instability (rapid change or death), the consumption of PC resources, mortality, and in-hospital death [9,10,11]. Identifying palliative complexity allows for the consistent adjustment of referrals to multidisciplinary PC teams and the intensity of their shared intervention with referring teams.

Ethical issues were identified as a variable with high predictive power for the level of palliative complexity. The data provided by this aggregated analysis confirm that the probability of high palliative complexity is significantly higher in patients presenting ethical issues.

### 4.4. Study Limitations

The PALCOM scale, the source of the data for this study, was designed for adult patients with advanced cancer. Therefore, the results are not generalizable to patients with non-oncological chronic diseases or to the pediatric population.

This study was carried out in a public healthcare system that provides universal access to all citizens, including palliative care services. It is unclear whether the observed data can be generalized to other healthcare settings.

## 5. Conclusions

This study confirms that ethical issues are highly prevalent in the end-of-life process of patients with advanced cancer. Ethical issues related to information and proportionality in the shared decision-making process are the most frequent. The presence of ethical dilemmas directly influences the complexity of palliative care needs. The early identification and addressing of ethical issues reduce their frequency during patient follow-up. Most observed ethical issues are directly or indirectly associated with patient autonomy.

## 6. Practical Implications

The data provided by this study confirm that, in routine clinical practice, it is essential to systematically explore the individual value and meaning each patient attributes to their illness and to identify emerging ethical conflicts. The early identification and addressing of these issues can improve the outcomes of supportive care. In this context, communication skills and basic training in bioethics play a crucial role for all healthcare professionals attending to incurable and life-threatening conditions.

## 7. Implications for Research

Due to the high prevalence of ethical issues in the end-of-life process of patients with advanced cancer and their impact on palliative complexity in real clinical practice, we believe it would be interesting to design prospective studies specifically aimed at analyzing the prevalence of different ethical issues and the determining factors for their occurrence.

We do not know the impact of euthanasia regulation laws on the prevalence of patients expressing a consistent desire to hasten death. An update of the PALCOM study would allow us to determine if there are differences in the prevalence of the desire to hasten death between a period before and after the implementation of the euthanasia regulation law in our country.

## Figures and Tables

**Table 1:** PALCOM scale of complexity of palliative care needs for advanced cancer patients.

Would you be surprised if the patient died in the next 12 months? If the answer is no, the PALCOM instrument can determine the complexity of palliative care needs and allows managing the intervention of specialized Palliative Care teams.					
1. Is a high symptom burden detected? Presence of ≥5 chronic symptoms with at least a moderate intensity (Visual Analogue Scale or Numeric Ratting Scale ≥4/10) out of 10 systematically recorded symptoms:					
▪ Pain ▪ Anorexia	▪ Weakness ▪ Nausea-vomiting	▪ Constipation ▪ Dyspnoea or cough	▪ Insomnia ▪ Drowsiness	▪ Anxiety ▪ Sadness	▪ Others
2. Are there any markers of difficult pain control? Any of the following characteristics can lead to potentially difficult pain:					
▪ Neuropathic pain. ▪ Pain associated with a history of addiction to alcohol or other substances of abuse.		▪ Mixed pain (nociceptive and neuropathic) ▪ Pain associated with cognitive impairment.		▪ Breakthrough cancer pain. ▪ Pain associated with intense emotional distress.	
3. Is there functional impairment? Person who requires relevant assistance for activities of daily living. (e.g., Barthel index ≤ 60 or Karnofsky index ≤ 50–60%)					
4. Any socio-familial risk factors?					
▪ Absence of identified caregiver. ▪ Caregiver limitations due to advanced age, health problems, or socio-family or economic burdens.		▪ Minors or more than one member of the nuclear family who needs support. ▪ Risk of severe family burnout.		▪ Other complexity situations (social vulnerability, poverty, domestic violence, addiction of abuse substances…)	
5. Any ethical or existential conflict?					
▪ Conflicts related to information (denial, conspiracy silence,…) ▪ Healthcare team disagreement.		▪ Disagreement between patient/family and healthcare team. ▪ Loss of meaning in life or existential distress. ▪ Spiritual distress.		▪ Desire to advance death, demand for euthanasia or assisted suicide. ▪ Others…	
Each of these 5 domains is scored dichotomously, 0 absence or 1 presence of any of the variables, the sum, between 0 and 5, is the total score of the PALCOM scale.					
▪ Low complexity (0–1): Basic palliative care is recommended. Referring team to get back in contact if patient becomes more complex. In some cases, timely consultation with specialist palliative care may be needed for a comprehensive assessment or management of difficult isolated symptoms. ▪ Medium complexity (2–3): Specialised palliative care is systematically recommended (hospital teams, home support teams or palliative care services). ▪ High complexity (4–5): Intensive specialised palliative care is systematically recommended (teams in the hospital, support teams in the home or palliative care services).					

**Table 2:** Characteristics of patients according to the development and validation cohorts of the PALCOM scale and pooled data.

			Development Cohort	Validation Cohort		PALCOM Pooled Data
			N (%)	N (%)	*p*	N (%)
Total			324	283		607
Gender		Male	189 (58.0)	161 (56.9)	0.280	350 (57.7)
Age		(mean ± SD)	69 (SD ± 59–80)	71 (SD ± 59–81)	0.320	70 (SD ± 59–80)
Primary origin						
	Lung		71 (21.9)	64 (24.4)	0.310	140 (23.1)
	Colon		38 (11.7)	56 (19.8)	0.290	94 (15.5)
	Pancreas		28 (8.6)	18 (6.4)	0.270	46 (7.6)
	Breast		22 (6.8)	19 (6.7)	0.190	41 (6.8)
	Prostate		18 (5.5)	28 (9.9)	0.210	46 (7.6)
	Others		146 (45.1)	93 (32.8)	0.300	239 (39.4)
Symptom prevalence						
	Asthenia		299 (92.2)	269 (95.1)	0.210	568 (93.6)
	Anorexia		253 (78.1)	226 (79.9)	0.079	479 (78.9)
	Pain		245 (75.6)	245 (86.6)	0.100	490 (80.7)
	Nausea		110 (34.0)	68 (24.0)	0.080	178 (29.3)
	Constipation		202 (62.3)	162 (57.2)	0.110	364 (59.9)
	Dyspnea		149 (45.9)	111 (39.2)	0.220	260 (42.8)
	Insomnia		191 (58.9)	177 (62.5)	0.180	368 (60.6)
	Anxiety		238 (73.4)	184 (65.0)	0.090	422 (69.5)
	Sadness		225 (69.4)	196 (69.3)	0.220	421 (69.4)
PALCOM domains						
	High symptom burden		134 (41.3)	136 (48.1)	0.190	270 (44.5)
	Refractory pain		175 (54.0)	166 (58.5)	0.220	341 (56.2)
	Karnofsky index ≤ 60%		129 (39.9)	135 (47.5)	0.210	264 (43.5)
	Socio-familial risk		221 (68.3)	184 (64.8)	0.420	405 (66.7)
	Ethical issues		59 (18.3)	67 (23.6)	0.080	126 (20.7)

**Table 3:** PALCOM scale domains according to the level of complexity of palliative care needs.

			Development Cohort				Validation Cohort				Pooled Data			
N (%)			324 (53.4% of polled data)				283 (46.6% of polled data)				607			
Hospital inclusion			180 (55.6)					175 (61.8)				355 (58.5)		
Community inclusion			144 (44.4)					108 (38.2)				252 (41.5)		
			Low	Medium	High		Low	Medium	High		Low	Medium	High	
			51 (15.8)	139 (43.0)	133 (41.2)		67 (23.7)	167 (59.9)	49 (17.3)		118 (19.5)	306 (50.5)	182 (30.0)	
PALCOM domains						*p*				*p*				*p*
	High symptom burden		20 (39.2)	99 (71.2)	115 (86.5)	<0.001	6 (8.9)	83 (49.7)	47 (95.5)	<0.001	26 (22.0)	182 (59.4)	162 (89.0)	<0.001
	Refractory pain		13 (25.5)	69 (49.6)	93 (69.9)	<0.001	30 (45.7)	93 (55.4)	43 (87.8)	<0.001	43 (36.4)	162 (52.9)	136 (74.7)	<0.001
	Karnofsky index ≤ 60%		17 (33.3)	53 (38.1)	59 (44.4)	<0.001	4 (5.9)	90 (53.6)	41 (83.7)	<0.001	21 (17.8)	143 (46.7)	100 (54.9)	<0.001
	Socio-familial risk		33 (64.7)	81 (58.3)	107 (80.5)	0.055	13 (19.4)	126 (75.0)	45 (91.8)	<0.001	46 (42.5)	207 (67.6)	152 (83.5)	<0.001
	Ethical issues		1 (2.0)	25 (18.0)	33 (24.8)	<0.001	4 (5.9)	34 (20.2)	29 (59.2)	<0.001	5 (4.2)	59 (19.5)	56 (30.8)	<0.001
Death within 6 months			18 (35.3)	77 (55.4)	99 (74.3)	<0.001	33 (49.2)	114 (68.3)	38 (77.6)	<0.001	51 (43.2)	190 (62.7)	138 (75.8)	<0.001
Hospital death			3 (5.9)	24 (17.3)	40 (30.1)	<0.001	6 (8.9)	25 (22.1)	12 (30.8)	<0.001	9 (7.6)	49 (16.0)	52 (28.6)	<0.001

**Table 4:** Frequency of specific ethical issues.

				PALCOM Development n (%)	PALCOM Validation n (%)	*p*	Pooled Data
Total				324 (53.4)	283 (46.6)	-	607
N patients with ≥1 ethical issue				59 (18.3)	67 (23.7)	0.419	126 (20.7)
Specific ethical issues							
-	Information			51 (15.7)	28 (10.0)	0.071	79 (13.0)
-	Proportionality of therapeutic or support measures			54 (16.7)	41 (14.5)	0.580	95 (15.6)
	*		Discrepancies within the family circle	26 (8.0)	21 (7.4)	0.879	47 (7.7)
	*		Discrepancies between the care team and patient or family	14 (4.3)	13 (4.6)	0.890	27 (4.4)
	*		Discrepancies within the care team	12 (3.7)	13 (4.6)	0.684	25 (4.1)
	*		Discrepancies with respect to withdrawal life support	2 (0.6)	1 (0.3)	0.900	3 (0.5)
-	Research and clinical trials			8 (2.5)	10 (3.5)	0.482	18 (2.9)
-	Request to hasten death			4 (1.2)	7 (2.4)	0.363	11 (1.8)
-	Conflicts associated with palliative sedation			0	1 (0.3)	0.257	1 (0.15)
Total ethical issues				117	87	0.202	204
Ethical issues/patient				1.9	1.3	0.202	1.6

## Data Availability

The data can be shared upon request.

## References

[1] Reich W.T. (1995). Introduction. Encyclopedia of Bioethics.

[2] Sallnow L., Smith R., Ahmedzai S.H., Bhadelia A., Chamberlain C., Cong Y., Doble B., Dullie L., Durie R., Lancet Commission on the Value of Death (2022). Report of the Lancet Commission on the Value of Death: Bringing death back into life. Lancet.

[3] Martin-Rosello M.L., Sanz-Amores M.R., Salvador-Comino M.R. (2018). Instruments to evaluate complexity in end-of-life care. Curr. Opin. Support. Palliat. Care.

[4] Grant M., de Graaf E., Teunissen S. (2021). A systematic review of classifications systems to determine complexity of patient care needs in palliative care. Palliat. Med..

[5] Grant M.P., Philip J.A.M., Deliens L., Komesaroff P.A. (2022). Understanding Complexity in Care: Opportunities for Ethnographic Research in Palliative Care. J. Palliat. Care..

[6] Glouberman S., Zimmerman B. (2002). Complicated and complex systems: What would successful reform of Medicare look like?. Rom. Pap..

[7] Munday D.F., Johnson S.A., Griffiths F.E. (2003). Complexity theory and palliative care. Palliat. Med..

[8] Hodiamont F., Jünger S., Leidl R., Maier B.O., Schildmann E., Bausewein C. (2019). Understanding complexity—The palliative care situation as a complex adaptive system. BMC Health Serv. Res..

[9] Tuca A., Gómez-Martínez M., Prat A. (2017). Predictive model of complexity in early palliative care: A cohort of advanced cancer patients (PALCOM study). Support. Care Cancer.

[10] Viladot M., Gallardo-Martínez J.-L., Hernandez-Rodríguez F., Izcara-Cobo J., Majó-Llopart J., Peguera-Carré M., Russinyol-Fonte G., Saavedra-Cruz K., Barrera C., Chicote M. (2023). Validation Study of the PALCOM Scale of Complexity of Palliative Care Needs: A Cohort Study in Advanced Cancer Patients. Cancers.

[11] Tuca A., Viladot M., Carrera G., Llavata L., Barrera C., Chicote M., Marco-Hernández J., Padrosa J., Zamora-Martínez C., Grafia I. (2024). Evolution of Complexity of Palliative Care Needs and Patient Profiles According to the PALCOM Scale (Part Two): Pooled Analysis of the Cohorts for the Development and Validation of the PALCOM Scale in Advanced Cancer Patients. Cancers.

[12] Cerullo G., Videira-Silva A., Carrancha M., Rego F., Nunes R. (2023). Complexity of patient care needs in palliative care: A scoping review. Ann. Palliat. Med..

[13] Tuca A., Viladot M., Barrera C., Chicote M., Casablancas I., Cruz C., Font E., Marco-Hernández J., Padrosa J., Pascual A. (2021). Prevalence of ethical dilemmas in advanced cancer patients (secondary analysis of the PALCOM study). Support. Care Cancer.

[14] Beauchamp T., Childress J. (2001). Moral norms. Principles of Biomedical Ethics.

[15] Ong W.Y., Yee C.M., Lee A. (2012). Ethical dilemmas in the care of cancer patients near the end of life. Singap. Med. J..

[16] Chih A.H., Su P., Hu W.Y., Yao C.A., Cheng S.Y., Lin Y.C., Chiu T.Y. (2016). The changes of ethical dilemmas in palliative care. A lesson learned from comparison between 1998 and 2013 in Taiwan. Medicine.

[17] Huang H.L., Yao C.A., Hu W.Y., Cheng S.Y., Hwang S.J., Chen C.D., Lin W.Y., Lin Y.C., Chiu T.Y. (2018). Prevailing ethical dilemmas encountered by physicians in terminal cancer care changed after the enactment of the Natural Death Act: 15 years’ follow-up survey. J. Pain Symptom. Manag..

[18] Guevara-López U., Altamirano-Bustamante M.M., Viesca-Treviño C. (2015). New frontiers in the future of palliative care: Real-world bioethical dilemmas and axiology of clinical practice. BMC Med. Ethics.

[19] Chiu T.-Y., Hu W.-Y., Huang H.-L., Yao C.-A., Chen C.-Y. (2009). Prevailing Ethical Dilemmas in Terminal Care for Patients with Cancer in Taiwan. J. Clin. Oncol..

[20] Kinzbrunner B.M. (1995). Ethical dilemmas in hospice and palliative care. Support. Care Cancer.

[21] Blanco Portillo A., García-Caballero R., Real de Asúa D., Olaciregui Dague K., Márquez Mendoza O., Valdez P., Herreros B. (2024). What ethical conflicts do internists in Spain, México and Argentina encounter? An international cross-sectional observational study based on a self-administrated survey. BMC Med. Ethics.

[22] Cherny N.I., Ziff-Werman B. (2023). Ethical considerations in the relief of cancer pain. Support Care Cancer.

[23] Crico C., Sanchini V., Casali P.G., Pravettoni G. (2022). Ethical issues in oncology practice: A qualitative study of stakeholders’ experiences and expectations. BMC Med. Ethic.

[24] Hui D., Bruera E. (2017). The Edmonton Symptom Assessment System 25 Years Later: Past, Present, and Future Developments. J. Pain Symptom Manag..

[25] Hui D., Bruera E. (2014). A Personalized Approach to Assessing and Managing Pain in Patients with Cancer. J. Clin. Oncol..

[26] Fainsinger R.L., Nekolaichuk C.L. (2008). A “TNM” classification system for cancer pain: The Edmonton Classification System for Cancer Pain (ECS-CP). Support. Care Cancer.

[27] Fainsinger R.L., Nekolaichuk C.L., Lawlor P.G., Neumann C.M., Hanson J., Vigano A. (2005). A Multicenter Study of the Revised Edmonton Staging System for Classifying Cancer Pain in Advanced Cancer Patients. J. Pain Symptom Manag..

[28] Gilligan T., Coyle N., Frankel R.M., Berry D.L., Bohlke K., Epstein R.M., Finlay E., Jackson V.A., Lathan C.S., Loprinzi C.L. (2017). Patient-Clinician Communication: American Society of Clinical Oncology Consensus Guideline. J. Clin. Oncol..

[29] Katz S.J., Belkora J., Elwyn G. (2014). Shared decision making for treatment of cancer: Challenges and opportunities. J. Oncol. Pract..

[30] Witteman H.O., Julien A.S., Ndjaboue R., Exe N.L., Kahn V.C., Fagerlin A., Zikmund-Fisher B.J. (2020). What Helps People Make Values-Congruent Medical Decisions? Eleven Strategies Tested across 6 Studies. Med. Decis. Mak..

[31] Ong L.M., Visser M.R., Lammes F.B., De Haes J.C. (2000). Doctor-patient communication and cancer patients’ quality of life and satisfaction. Patient Educ. Couns..

[32] Zachariae R., Pedersen C.G., Jensen A.B., Ehrnrooth E., Rossen P.B., von der Maase H. (2003). Association of perceived physician communication style with patient satisfaction, distress, cancer-related self-efficacy, and perceived control over the disease. Br. J. Cancer.

[33] Lorusso D., Bria E., Costantini A., Di Maio M., Rosti G., Mancuso A. (2017). Patients’ perception of chemotherapy side effects: Expectations, doctor-patient communication and impact on quality of life—An Italian survey. Eur. J. Cancer Care.

[34] Pozzar R.A., Xiong N., Hong F., Wright A.A., Goff B.A., Underhill-Blazey M.L., Tulsky J.A., Hammer M.J., Berry D.L. (2021). Perceived patient-centered communication, quality of life, and symptom burden in individuals with ovarian cancer. Gynecol. Oncol..

[35] Mack J.W., Fasciano K.M., Block S.D. (2018). Communication About Prognosis with Adolescent and Young Adult Patients with Cancer: Information Needs, Prognostic Awareness, and Outcomes of Disclosure. J. Clin. Oncol..

[36] Shin J.A., El-Jawahri A., Parkes A., Schleicher S.M., Knight H.P., Temel J.S. (2016). Quality of Life, Mood, and Prognostic Understanding in Patients with Metastatic Breast Cancer. J. Palliat. Med..

[37] Lin J.J., Chao J., Bickell N.A., Wisnivesky J.P. (2016). Patient-provider communication and hormonal therapy side effects in breast cancer survivors. Women Health.

[38] Jacobs J.M., Pensak N.A., Sporn N.J., MacDonald J.J., Lennes I.T., Safren S.A., Pirl W.F., Temel J.S., Greer J.A. (2017). Treatment Satisfaction and Adherence to Oral Chemotherapy in Patients with Cancer. J. Oncol. Pract..

[39] Albrecht T.L., Eggly S.S., Gleason M.E., Harper F.W., Foster T.S., Peterson A.M., Orom H., Penner L.A., Ruckdeschel J.C. (2008). Influence of Clinical Communication on Patients’ Decision Making on Participation in Clinical Trials. J. Clin. Oncol..

[40] Weeks J.C., Catalano P.J., Cronin A., Finkelman M.D., Mack J.W., Keating N.L., Schrag D. (2012). Patients’ Expectations about Effects of Chemotherapy for Advanced Cancer. N. Engl. J. Med..

[41] Lobb E.A., Halkett G.K., Nowak A.K. (2011). Patient and caregiver perceptions of communication of prognosis in high grade glioma. J. Neurooncol..

[42] Liu P.-H., Landrum M.B., Weeks J.C., Huskamp H.A., Kahn K.L., He Y., Mack J.W., Keating N.L. (2014). Physicians’ Propensity to Discuss Prognosis Is Associated with Patients’ Awareness of Prognosis for Metastatic Cancers. J. Palliat. Med..

[43] Tang S.T., Wen F.-H., Hsieh C.-H., Chou W.-C., Chang W.-C., Chen J.-S., Chiang M.-C. (2016). Preferences for Life-Sustaining Treatments and Associations with Accurate Prognostic Awareness and Depressive Symptoms in Terminally Ill Cancer Patients’ Last Year of Life. J. Pain Symptom Manag..

[44] Graham J., Ramirez A. (2002). Improving the working lives of cancer clinicians. Eur. J. Cancer Care.

[45] Cherny N., Nortjé N., Kelly R., Zimmermann C., Jordan K., Kreye G., Le N.-S., Adelson K. (2025). A taxonomy of the factors contributing to the overtreatment of cancer patients at the end of life. What is the problem? Why does it happen? How can it be addressed?. ESMO Open.

[46] Chen A.B., Cronin A., Weeks J.C., Chrischilles E.A., Malin J., Hayman J.A., Schrag D. (2013). Expectations About the Effectiveness of Radiation Therapy Among Patients with Incurable Lung Cancer. J. Clin. Oncol..

[47] Epstein A.S., Prigerson H.G., O’reilly E.M., Maciejewski P.K. (2016). Discussions of Life Expectancy and Changes in Illness Understanding in Patients with Advanced Cancer. J. Clin. Oncol..

[48] Enzinger A.C., Zhang B., Schrag D., Prigerson H.G. (2015). Outcomes of Prognostic Disclosure: Associations with Prognostic Understanding, Distress, and Relationship with Physician Among Patients with Advanced Cancer. J. Clin. Oncol..

[49] Roll I.J., Simms V., Harding R. (2009). Multidimensional Problems Among Advanced Cancer Patients in Cuba: Awareness of Diagnosis Is Associated with Better Patient Status. J. Pain Symptom Manag..

[50] Mystakidou K., Tsilika E., Parpa E., Katsouda E., Vlahos L. (2005). Patterns and barriers in information disclosure between health care professionals and relatives with cancer patients in Greek society. Eur. J. Cancer Care.

[51] Corbett V., Epstein A.S., McCabe M.S. (2018). Characteristics and Outcomes of Ethics Consultations on a Comprehensive Cancer Center’s Gastrointestinal Medical Oncology Service. HEC Forum.

[52] Bernat J.L. (2005). Medical Futility: Definition, Determination, and Disputes in Critical Care. Neurocritical Care.

[53] Rolak S., Elhawary A., Diwan T., Watt K.D. (2024). Futility and poor outcomes are not the same thing: A clinical perspective of refined outcomes definitions in liver transplantation. Liver Transplant..

[54] AMA Code of Medical Ethics Opinion 2.1.3. https://code-medical-ethics.ama-assn.org/opinions.

[55] Code of Medical Ethics (2016). Organización Médica Colegial de España. https://www.cgcom.es.

[56] Foster C. (2010). Autonomy should chair, not rule. Lancet.

[57] Guindalini R.S.C., Riechelmann R.P., Arai R.J. (2019). Personalizing Precision Oncology Clinical Trials in Latin America: An Expert Panel on Challenges and Opportunities. Oncologist.

[58] Daugherty C.K. (1999). Ethical Issues in the Development of New Agents. Investig. New Drugs.

[59] Schaeffer M.H., Krantz D.S., Wichman A., Masur H., Reed E., Vinicky J.K. (1996). The impact of disease severity on the informed consent process in clinical research. Am. J. Med..

[60] Daugherty C.K. (1999). Impact of Therapeutic Research on Informed Consent and the Ethics of Clinical Trials: A Medical Oncology Perspective. J. Clin. Oncol..

[61] Meropol N.J., Weinfurt K.P., Burnett C.B., Balshem A., Benson A.B., Castel L., Corbett S., Diefenbach M., Gaskin D., Li Y. (2003). Perceptions of Patients and Physicians Regarding Phase I Cancer Clinical Trials: Implications for Physician-Patient Communication. J. Clin. Oncol..

[62] Twycross R. (2019). Reflections on palliative sedation. Palliat. Care Res. Treat..

[63] Materstvedt L.J. (2019). Distinction between euthanasia and palliative sedation is clear-cut. J. Med. Ethic.

[64] Cherny N.I., ESMO Guidelines Working Group (2014). ESMO Clinical Practice Guidelines for the management of refractory symptoms at the end of life and the use of palliative sedation. Ann. Oncol..

[65] Balaguer A., Monforte-Royo C., Porta-Sales J., Alonso-Babarro A., Altisent R., Aradilla-Herrero A., Bellido-Pérez M., Breitbart W., Centeno C., Cuervo M.A. (2016). An International Consensus Definition of the Wish to Hasten Death and Its Related Factors. PLoS ONE.

[66] Rodríguez-Prat A., Balaguer A., Booth A., Monforte-Royo C. (2017). Understanding patients’ experiences of the wish to hasten death: An updated and expanded systematic review and meta-ethnography. BMJ Open.

[67] Belar A., Arantzamendi M., Santesteban Y., López-Fidalgo J., Martinez M., Lama M., Rullán M., Olza I., Breeze R., Centeno C. (2020). Cross-sectional survey of the wish to die among palliative patients in Spain: One phenomenon, different experiences. BMJ Support. Palliat. Care.

[68] Rodríguez-Prat A., Pergolizzi D., Crespo I., Julià-Torras J., Balaguer A., Kremeike K., Voltz R., Monforte-Royo C. (2024). The Wish to Hasten Death in Patients with Life-Limiting Conditions. A Systematic Overview. J. Pain Symptom Manag..

[69] Bellido-Pérez M., Monforte-Royo C., Tomás-Sábado J., Porta-Sales J., Balaguer A. (2016). Assessment of the wish to hasten death in patients with advanced disease: A systematic review of measurement instruments. Palliat. Med..

[70] World Population Review Countries Where Euthanasia Is Legal in 2024. https://worldpopulationreview.com/country-rankings/where-is-euthanasia-legal.

[71] Ley Orgánica 3/2021, de 24 de Marzo, de Regulación de la Eutanasia. Ministerio de la Presidencia, Justicia y Relaciones con las Cortes. Gobierno de España. Boletín Oficial del Estado. núm. 72, de 25 de Marzo de 2021.. https://www.boe.es/buscar/act.php?id=BOE-A-2021-4628.

